# Comparative CFD Simulations of a Soft Robotic Fish for Undulatory Swimming Behaviors

**DOI:** 10.3390/biomimetics10120805

**Published:** 2025-12-02

**Authors:** Gonca Ozmen Koca, Mustafa Ay, Cafer Bal, Deniz Korkmaz, Zuhtu Hakan Akpolat

**Affiliations:** 1 Department of Mechatronics Engineering, Faculty of Technology, Firat University, 23200 Elazig, Turkey; mustafaay@firat.edu.tr (M.A.); cbal@firat.edu.tr (C.B.); 2 Department of Electrical and Electronics Engineering, Faculty of Engineering and Natural Sciences, Malatya Turgut Ozal University, 44200 Malatya, Turkey; deniz.korkmaz@ozal.edu.tr; 3 Department of Electrical-Electronics Engineering, Faculty of Engineering, Fatih Sultan Mehmet Vakif University, 34015 Istanbul, Turkey; zhakpolat@fsm.edu.tr

**Keywords:** robotic fish, soft robot, hydrodynamic analysis, Ansys fluent, CFD, prediction model, deep learning

## Abstract

Studies on autonomous underwater vehicles (AUVs) have gained momentum in recent years, and a special type of AUV, the robotic fish, has become a significant topic, with a superior maneuverability to traditional AUVs. In this paper, a prediction strategy for the hydrodynamic performance of a robotic fish to analyze undulatory swimming behaviors is proposed. The two-dimensional robotic fish model for computational fluid dynamics (CFD) simulations is constructed, and a dynamic network method is applied to orient the generated network based on the wavy motion. For the thrust force of the fin, a body traveling wave is derived. In the simulations, the effects of kinematic parameters such as flapping frequency and speed on swimming efficiency and drag are analyzed, and thrust force production, power expenditure, and overall efficiency of swimming are examined. Later, a deep learning-based prediction model is designed from the obtained parameters, and force predictions are performed. Long short-term memory (LSTM)-, convolutional neural network (CNN)-, and gated recurrent network (GRU)-based time series prediction models are used, and their variations are compared. In these experiments, while the CNN-GRU achieves the higher prediction performance for the root mean square error, with 0.0228, other approaches give a lower performance, between 0.0233 and 0.0359. The proposed method demonstrates a superior performance in CNN and LSTM models and exhibits lower prediction errors.

## 1. Introduction

Robotic systems that aim to solve engineering problems excite researchers in underwater studies about fish robotics and its applications. In this context, the hydrodynamic analysis of underwater robots inspired by the movement characteristics of real fish gain importance. To see the details of the mechanics of fish swimming, it becomes necessary to analyze the forces exerted by the water and how these forces affect the movement of the fish. With the development of soft robot applications, the hypothesis of whether the transition from rigid-body robots to soft-body robots gives more realistic results in imitating the movements of the inspired real living creature is a subject worth investigating.

Studies on the computational fluid dynamics (CFD) analysis of robotic fish include topics such as the hydrodynamic characteristics of different robotic fish designs, including body shape, fin configurations, and propulsion mechanisms; the interaction between the robotic fish’s fins and the surrounding fluid; the hydrodynamic principles underlying fish locomotion, incorporating them into robot design; flow control mechanisms employed by robotic fish to enhance performance and stability; and the impact of environmental factors such as currents, turbulence, and obstacles on the performance of robotic fish. In their study, Chen et al. presented an experimental investigation aimed at determining the most efficient fin structure by comparing the hydrodynamic propulsion performance of different biomimetic pectoral fin designs on a single robotic platform [1]. Zhang conducted a fluid–structure interaction (FSI) analysis of a soft biomimetic robotic fish driven by a piezoelectric MFC actuator to examine its hydrodynamic force, displacement, and thrust performance at different drive frequencies [2]. The study demonstrated that the finite element and dynamic mesh-based FSI simulations were consistent with the experimental results, indicating that the robotic fish’s behavior in a flow environment can be predicted and utilized for design and performance improvements. A kinematic model and RBF neural network-based dynamic control approach were developed to enable flexible-body robotic fish to perform a constant-radius turning motion. The effects of parameters such as body offset, maximum bending time, and tail fin frequency on turning performance were investigated through a CFD analysis [3]. Liu et al. developed a strategy to predict the hydrodynamic performance of body–caudal fin (BCF)-propelled biomimetic fish using a NACA0012-based two-dimensional (2D) swimming model and CFD simulations and examined the influence of parameters such as flow velocity, frequency, wavelength, and head oscillation amplitude [4]. The study demonstrates that a Multi-Layer Perceptron (MLP)-based prediction model trained on these CFD results showed a high accuracy (3% error) and good generalization ability. Khan et al. investigated the effects of fluid loading, fin membrane structure, and target object geometry on contact perception by examining the strain responses of compliant robotic fins and stringers during underwater contact. By comparing contact processes in air and water environments, the study revealed how underwater structure–fluid structure interaction (SFSI) alters contact perception [5]. A detailed motion analysis was presented using three-dimensional (3D) CFD to examine the effects of the distinctive body shape and high-amplitude undulations of frog larvae (tadpoles) on swimming hydrodynamics and propulsion efficiency [6]. In their study, Wang et al. presented an optimization method combining a Hopf-based improved CPG control network with CFD simulations to improve the swimming performance of a three-joint biomimetic soft robot fish [7]. By analyzing the effects of CPG parameters on complex swimming behavior using a CFD-based simulation platform, the study accelerated experimental optimizations. It achieved an optimized swimming performance that was highly consistent with real-world tests. The hydrodynamic propulsion mechanism of a floating robot inspired by the Black Ghost Knife Fish was investigated through numerical simulations and experiments, investigating the effects of parameters such as the fin ratio, frequency, amplitude, and wave number on thrust generation [8]. The study also revealed how propulsion performance decreases in choppy water compared to still water, providing systematic design guidance for selecting appropriate motion parameters for different missions. In addition, CFD simulations are important in evaluating hydrodynamic drag and thrust forces to prepare robotic fish for long-term missions by reducing energy consumption [9,10,11].

Soft robots, inspired by the locomotion of fish and other aquatic creatures, offer advantages in agility, flexibility, and adaptability compared to traditional rigid robots. Soft robots, many of which are inspired by biological organisms, can perform a wide variety of tasks due to their ability to change shape and adapt to different surfaces [12]. Soft robots mimic the structures and movements of the organisms they inspire and can thus gain extraordinary abilities, such as navigating confined spaces or passing through narrow gaps. With their deformable bodies, soft robots can more easily adapt to unpredictable environments, changes in their environment, or task requirements, and overcome obstacles more easily. Since they do not require complex joints and mechanisms, they have lower production costs with their simpler designs. With their simple structure, they can be more energy efficient than rigid robots. CFD analysis plays a crucial role in understanding the hydrodynamics of these soft robots and optimizing their design for efficient locomotion. Since soft robotic fish tend to have more flexible structures and more complex behaviors than rigid-bodied robots, CFD analysis contains many clues showing fluid–structure interactions for behavioral control [13].

In our previous work, we focused on a robotic system that can convert a multi-joint serial mechanism into wave-like undulations [14,15,16,17]. To overcome the mechanical limitations and excessive energy consumption caused by multiple joints, in this study, we present a design that can model wave-like undulations within an integrated body structure capable of achieving the tail motion of fish-like swimmers.

The research on the propulsive principle of underwater animals has become more significant for underwater robotic applications and attracted more researchers’ attention. To figure out the mechanics of fish swimming, one needs to know the forces performed by the fluid and how these forces influence the fish’s motion. Li et al. modeled a zebrafish larva, which has a soft body, with the 3D computational method and analyzed it for both cyclic and non-cyclic swimming modes. A comparison of CFD and the experiment was ensured by comparing the output of the model with the experimental flow and kinematic data. The authors tried to discover the response of the body wave amplitude on performance during cyclic swimming to discover ideal swimming strategies [18]. Palit et al. investigated the fluid flows by implementing a CFD model of a tilapia fish. This rigid-body model was simulated by generating a movement in the form of a sinusoidal wave in the (*x*, *y*) plane of the tail and abdomen. Pressure and velocity curve results have been presented in CFD analysis and discussed concerning the aerodynamic force parameters for the linear motion of fish [19]. Adkins et al. established a CFD simulation of a 3D biomimetic fish-like body to explore the fluid flows around the body in a viscous liquid. The analysis ensured by the transient state simulation of a fish-like body has allowed the flow surrounding a fish-like body undergoing periodic oscillations to be investigated [20].

In the study of Liang et al., the characteristics of fluid flow and contours of vorticity around a three-dimensional traveling wave undulation body for tuna in straight motion were investigated by developing a CFD model. Only the posterior half of the fish model was undulated, and the lunate fin oscillating together with a swaying with yawing mode was considered as the tail. The hydrodynamic performance of the fish body model was investigated for the effects of the interaction between the vortices of the body and the tail [21]. Cui et al. determined the movement index by separating the body of the carangiform fish from its tail using complex orthogonal decomposition. The swimming performance of the fish was examined, with different amplitudes and tail flapping frequencies, with a movement index of around 0.6. As a result of the study, it was revealed that forward speed is related to the movement index and flapping frequency, and swimming efficiency is related to the flapping frequency and amplitude coefficient, as seen in real fish [22]. Chang et al. analyzed thrust forces using crescent, semicircular, and fan-shaped fin models with CFD simulations. Turbulence effects were examined using two typical turbulence models (SA, SST) [23].

There are many hypotheses to investigate the behavioral characteristics and functionality of fish schools. The escape reactions of schooling fish against approaching predators spread from the first reaction to the following individuals, resulting in changes in orientation and swimming speed, which help the individuals to escape quickly. In their study, Takagi et al. measured the 3D positions of individuals in a school in a largely closed environment in order to understand the connection between the alignment of individuals in the school and energy saving. Restricted swimming trials have shown that, at peak flow rates, the tail beat frequency (TBF) of shoal followers is significantly lower than that of pioneer fish, and the hydrodynamic effects on this were investigated in the study [24]. Researchers have determined that fish in nature behave in ways that minimize their energy consumption while swimming. In order to understand these behavioral models, Eguchi et al. tried to simulate the swimming behavior of fish in which they use the stagnation area of the flat plate to stay in their current position. The study attempted to show that fish adjust the forces applied to their bodies by changing the curvature of their tail fins, which is necessary to stay in place relative to the flow field. It is predicted that this study may be effective in ensuring fish schooling behavior in future studies [25].

Park and Sung modeled a flexible fin by examining the hydrodynamic effects experienced by fish in irregular flows and tried to reveal the mechanisms by which fish can use energy from the behavior of the liquid around them. To do this, they examined three different irregular flow situations: close to the ground, behind the cylinder, and behind a moving fin. It is stated that the study will contribute not only to understanding the unique behavior of fish in perturbed flows but also to the development of artificial underwater vehicles that benefit from environmental energy [26]. Curatolo and Teresi aimed to model muscle functioning using the active distortion system. In the study, kinematic behaviors were examined rather than realizing the basic characteristics of carangiform swimming [27]. Chang et al. studied the effect of the Reynolds number on the thunniform mode of fish. The dynamic hybrid mesh method and unsteady incompressible flow solver were used in the simulation model. Increasing the Reynolds number caused the surface friction coefficient to decrease. Additionally, the study concluded that it is appropriate for thunniform fish to swim at higher speeds at higher Reynolds numbers. The performances of three different shaped tail fin models, namely crescent-shaped, semicircle-shaped, and fan-shaped, were also tested [23].

Macias et al. examined the hydrodynamics of the wake flow behind two fish of the carangiform species. It was simulated by allowing fish to move in laminar and turbulent flows. The patterns revealed by the swimming traces of fish were examined, and the relationships between these traces and the kinematics of the fish were studied [28]. Costa et al. designed a series of ostasiiform swimming robots and designed a transmission system that converts the continuous rotation motion of a single motor into the undulating motion of a multi-joint serial mechanism that will benefit from maximum thrust while maintaining the fish’s maneuverability to a certain extent. The propulsive performance of the caudal fin was examined using CFD techniques. The robotic propulsion performance was analyzed dynamically. The authors stated that their work is still in the prototyping stage [29]. Xia et al. carried out the hydrodynamic analysis of porpoise movement in dolphins in a model with a dolphin-like body. For this purpose, a four-stage swimming model was used: underwater acceleration, jumping up, gliding in the air, and diving down. In the study, the maximum jump height, maximum jump distance, and jump efficiency were simulated for various behaviors by changing the flapping frequency and escape angle [30].

In recent years, studies on biomimetic underwater robots have moved beyond fin or body oscillation (BCF) locomotion to propulsion mechanisms using alternative soft-body structures. In this context, Hu et al. [31] developed a jet-propelled soft robotic jellyfish inspired by the origami polyhedral structure and reported that CFD simulations and experimental results showed a high agreement. This study constitutes a significant example in the literature, demonstrating the effectiveness of structural deformation-based design approaches in underwater propulsion mechanisms [31].

For the above existing CFD simulations of fish studies, there are still some open issues. The deep analysis of wake flows using intelligent methods may hold more clues about fish hydrodynamics. In certain cases where studies are available in the literature, classifying fish movements through CFD analysis will also contribute to energy-saving underwater robotic applications. Further research applying deep learning methods is needed to reveal the effects of hydrodynamic data obtained through CFD analysis, depending on the swimming model and boundary conditions, on swimming performance.

To more clearly present the position of this study within the existing literature and to highlight the novelty of the proposed approach, a comparative table summarizing the representative CFD–machine learning studies and CFD-assisted biomimetic fish research utilized in the article is provided (Table 1).

In this study, a deep learning-based approach is proposed to model and predict the undulatory swimming behavior of a robotic fish. A 2D robotic fish model is implemented within a CFD framework to reduce computational costs while preserving physical accuracy. A user-defined function (UDF) is developed to capture the fish-like wavy motion of the model in the CFD environment. We enhance simulation flexibility by integrating this UDF into our CFD framework, thereby providing customized force calculations and adaptive boundary conditions to capture the complexity of fish swimming dynamics better. Based on the data obtained from these simulations, a CNN-GRU hybrid deep learning model is designed to effectively extract distinctive features related to swimming dynamics. The proposed model demonstrated a high accuracy in predicting hydrodynamic forces and swimming performance, achieving significant improvements in error metrics such as *RMSE*, *MAE*, and *SMAPE*. Furthermore, the model outputs are evaluated using performance coefficients, including *C_T_*, *C_P_*, and *η*, to thoroughly investigate the effects of various kinematic parameters on swimming efficiency. Overall, the study presents a time-efficient and accurate deep learning-assisted solution compared to traditional CFD-based methods, offering valuable insights for both biological studies and engineering applications.

The remaining parts of this paper are organized as follows: Section 2 presents the details of background theories. The experiments, findings of the study, and performance comparisons are given in Section 3. Finally, Section 4 summarizes the conclusions of the study.

## 2. Materials and Methods

### 2.1. Numerical Setup

Liu et al. [6] analyzed tadpole propulsion by employing a 3D CFD model and compared the outcomes with those obtained from a previously conducted 2D study. Their findings indicated that the 3D simulations validated the results of the earlier 2D analysis. The 3D geometries of biomimetic fish-like bodies are often highly complex. The processes of mesh generation and the accurate specification of boundary conditions for such geometries are both time-consuming and prone to error. Moreover, 3D CFD simulations require substantially greater computational power, memory capacity, and processing time. This is particularly the case in simulations involving detailed turbulence modeling and time-dependent analyses, where 3D solutions can become prohibitively expensive. In contrast, 2D models are simpler and more manageable, providing sufficient insights into the general flow behavior and fundamental aerodynamic/hydrodynamic characteristics.

**Figure 1:**
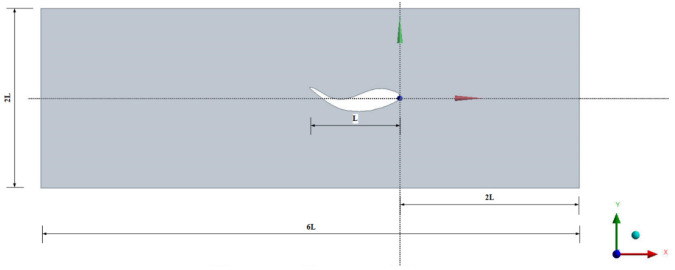
Scaled fish geometry and flow boundary.

A 2D model is used to accurately simulate the undulating motion of a fish-like body and the flapping of the tail fin. As shown in Figure 1, the total length of the fish model is *L* = 1 m, while the computational domain used has dimensions of 2*L* × 6*L*, and the fish model is placed 2*L* to the right of the input.

**Figure 2:**
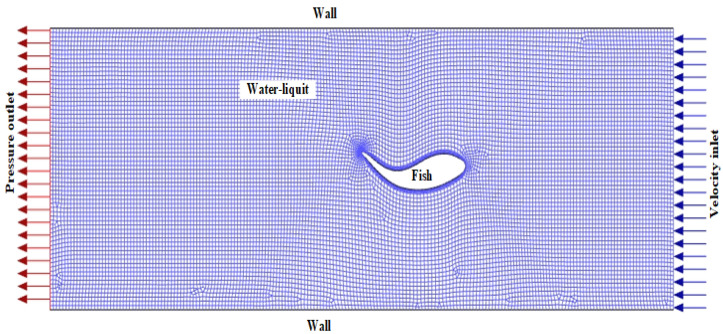
Mesh scheme and flow boundary conditions.

A comprehensive view of the mesh used in the liquid areas and around the fish is presented in Figure 2. Ansys Fluent 21, a commercial CFD software, is used to solve the control equations throughout the computational domain. Hexa meshes are used to achieve faster and better solutions in the liquid areas. A five-layer, 1.2-layer thickness increment inflation is applied to the 2D fish model boundary layer to accurately analyze the flow behavior in the boundary regions. Throughout the simulation, dynamic mesh-based smoothing, layering, and remeshing are applied to maintain the quality of the mesh structure and ensure its deformation and reconstruction during the flexible deformation motion of the fish body during its wavy motion. In carangiform and thunniform swimmers, the body is characterized by undulations backwards from the tail. The kinematics of this movement are described using a sinusoidal equation for the transverse displacement of the midline of the fish, as reported by many researchers [20,21,22]:

(1) $$h\left(x,t\right)=a\left(x\right)\sin\left(kx-\omega t\right)$$

In Equation (1), *ω* = 2*π f* (rad/s) is the swimming frequency, *k* = 2*π*/*λ* is the tail wave number, *λ* represents the wavelength, and *a*(*x*) is the variable amplitude, defined by Equation (2):

(2) $$a\left(x\right)=a_{0}+a_{1}x+a_{2}x^{2}$$

With the kinematic model describing the tail motion (Equations (1) and (2)), a FLUENT user-defined function (UDF) is created to track the instantaneous position and state of the tail, where *a*_0_ = 0.2, *a*_1_ = 0.01, and *a*_2_ = 0.1.

The generated UDF algorithm is a separate function written in the C programming language. This function is dynamically loaded into the FLUENT solver. In this study, a dynamic mesh UDF is implemented using the DEFINE_GRID_MOTION macro to simulate fish movement.

In this study, the flow is set in the form of the motion of a 2D body deforming in the form of undulation in an incompressible viscous fluid. The corresponding governing equations, the Navier–Stokes equations written for the incompressible viscous flow of a Newtonian fluid with a constant density and kinematic viscosity, are solved using computational fluid dynamics (CFD). The SIMPLE (Semi Implicit Pressure Linked Equations) algorithm is used as the coupling scheme of the pressure–velocity equations. The SST k-ω turbulence model (Shear Stress Transport model) is an advanced turbulence model used to accurately analyze near-wall flows and free-stream regions simultaneously. In this study, the SST k-ω model is adopted to accurately capture the vortex dynamics in the flow under different flow regimes and to calculate the hydrodynamic forces that play a role in the driving motion. In the simulation with dynamic motion, the time step is chosen to be 0.001 s. The analysis of the numerical analysis described in detail is performed on a workstation with a 128 Core 256 Processor AMD EPYC 7763 CPU @2.45 GHz.

Most studies of carangiform and thunniform swimmers have emphasized two non-dimensional parameters associated with swimming performance. The Reynolds number (*Re*) of the flow, the Strouhal number (*St*) depending on the frequency, and the amplitude of the undulating body movement are defined as follows [23,24].

(3) $$Re=\frac{UL}{\nu }$$

where *L* is the fish length, *U* is the flow velocity, and *ν* is the kinematic viscosity of the fluid (water).

(4) $$St=\frac{fA}{U}$$

where *f* is frequency and *A* is the amplitude of fish tail. The Reynolds number is set by changing the flow velocity of the fluid, while the Strouhal number is determined by changing the frequency of the swimming motion, such that both *Re* and *St* can be set independently. In this study, simulations are carried out considering the fish as being steady in a constant velocity flow. In this study, the non-dimensional parameters *Re* and *St*, which are associated with swimming performance, are varied over a systematic range to investigate measures of efficiency.

### 2.2. Sensitivity of Grid

**Figure 3:**
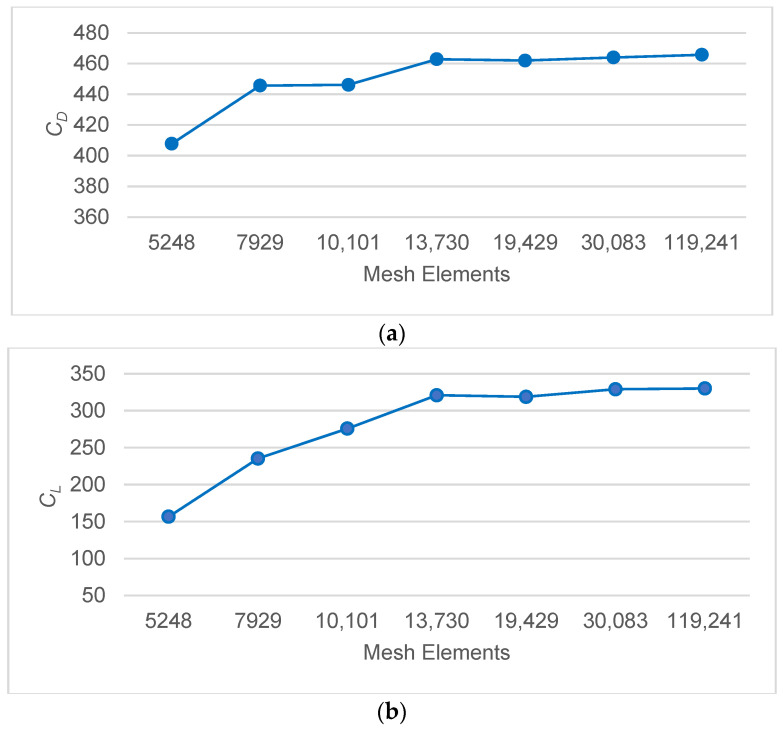
Parametric analysis for mesh sensitivity for (**a**) *C_D_* and (**b**) *C_L_*.

It is known that the density and quality of the mesh have a great influence on the convergence of the results obtained in CFD simulations. In order to determine the ideal mesh density in our simulation model, a parametric study is performed in an Ansys fluent environment. While creating the mesh diagram of the computational domain, the size of the mesh grid is selected as a parameter variable. The fish body oscillation frequency *f* = 2 Hz, flow velocity *V* = 0.2 m/s, and SST k-ω SST turbulence model are used in the simulation. Then, the drag force coefficient *C_D_* criteria are adopted to determine the convergence of each simulated operating condition. The data obtained as a result of the parametric study are given in Table 2 and Figure 3.

As seen in Figure 3, M4, M5, and M6 meshes with different numbers of elements did not cause a significant change in the *C_D_* value. In addition, the orthogonal quality mesh measurements are highest in the M4 mesh structure, with 0.73. This mesh size will give better results in a shorter computational time. In light of this data, all subsequent simulations are performed using the M4 mesh structure.

**Figure 4:**
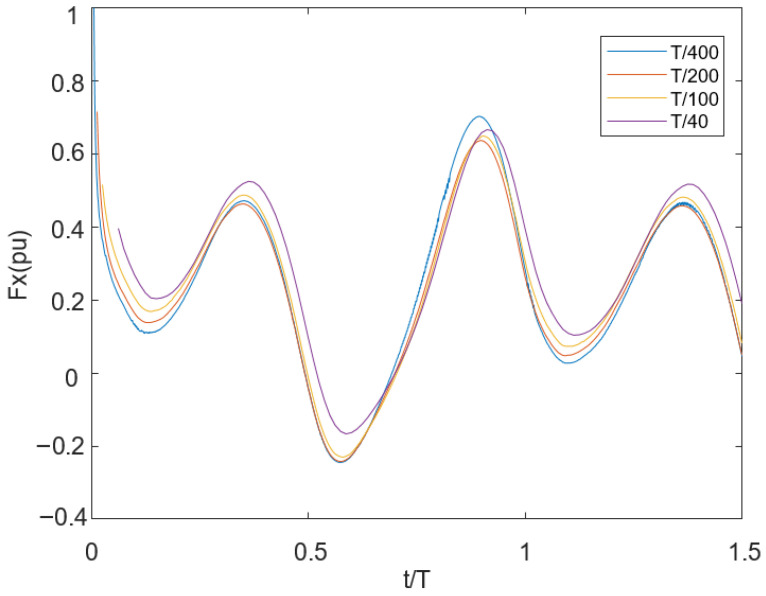
*Fx* results for time step convergence study (four different time steps *Δt*/*T* ∈ [1/40, 1/100, 1/200, 1/400]).

The increase in buoyancy observed in the M2 → M3 transition is due to the more sensitive capture of velocity gradients, particularly thanks to the application of a five-layer, 1.2 incrementinflation mesh structure applied to the upper and lower surfaces of the fish body. The local densification of cells in the M3 mesh structure enabled the resolution of sharper pressure gradients, particularly in the separation zone on the underside of the body, leading to an increase in *C_L_*. In contrast, because drag is a more global force acting on the entire body, *C_D_* is less affected by the mesh change. As reported in the literature, since buoyancy is more sensitive to local pressure differences, this limited improvement in mesh quality has a noticeable effect on *C_L_*. Still, it does not result in a significant change in *C_D_*. Figure 4 shows the convergence results of the time step size.

In the time step convergence study, the *Fx* results obtained from the tail motion simulation with a flow velocity of 0.1 m/s and a frequency of 0.5 Hz are compared using four different time steps: *Δt*/*T* ∈ [1/40, 1/100, 1/200, 1/400]. Here, it is observed that *Δt*/*T* = 1/200 and 1/400 yielded temporally convergent results, with an error of 2.99%, while the error rate between 1/400 and 1/100 was approximately 4.06%. While it is true that theoretically choosing a small time step would be beneficial for increasing the solution accuracy, a very small time step would increase the amount of computation and reduce the solution efficiency. As a result, the *Δt* = *T*/100 time step is used in the simulations of our model, since a better solution accuracy and efficiency can be achieved.

### 2.3. Validation of Numerical Method

**Figure 5:**
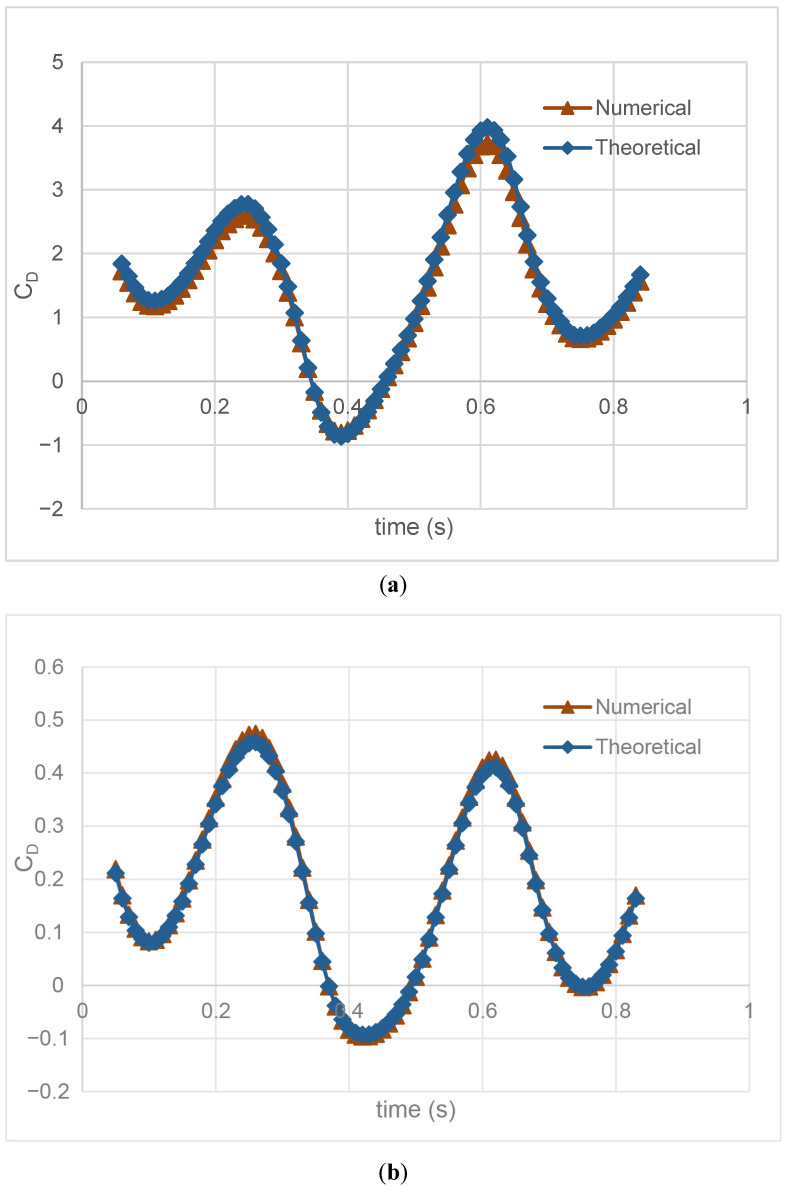
Comparison between the numerical and theoretical thrust force coefficients (*C_D_*) for (**a**) flow velocity *U* = 0.2 m/s and (**b**) *U* = 0.5 m/s.

In Figure 5, the drag force coefficient is presented for approximately one period at flow speeds of *U* = 0.2 m/s and *U* = 0.5 m/s. The flapping frequency is determined as 1.5 Hz for both results. Although the numerical and theoretical results are quite consistent, a deviation of 5% occurs for *U* = 0.2 m/s and 2% for *U* = 0.5 m/s. The acceptable error rate observed in theoretical and simulation results shows the validity of the numerical model methodology created for fish.

### 2.4. Calculation of Hydrodynamic Forces and Efficiency

In undulatory swimming, the propulsive body wave traverses the fish body from head to tail fin at a speed greater than the overall swimming speed [32]. The fish model with a soft body applied in this study makes the oscillation that provides the swimming movement with its entire body. The fish body’s oscillation movements must cope with the strength of the fluid surrounding it. The robot’s forward motion is obtained as the result of the drag in the *x*-direction and the thrust force in the opposite direction. The thrust force coefficient, which is a dimensionless parameter obtained as a measure of the thrust force acting on the robotic fish as it moves in the fluid, is given in Equation (5).

(5) $$C_{x}\left(t\right)=\frac{2F_{x}\left(t\right)}{\rho U^{2}}$$

where *Fx*(*t*) is the thrust force, *ρ* is the density (998.2 kg/m^3^), and *U* is the flow velocity. The average thrust force coefficient in a period is expressed as *C_T_* as follows:

(6) $$C_{T}=\frac{1}{T}\int_{0}^{T}C_{x}\left(t\right)dt$$

We also can describe the drag coefficient as *C_D_* = −*C_T_*. The input power transferred to create the oscillating motion in the fish body is used to cope with the hydrodynamic forces on the foil (*F*). The input power (*P_in_*) can be expressed as below with Equation (7).

(7) $$P_{in}=\frac{1}{T}\int_{0}^{T}F\left(t\right)\dot{h}\left(x,t\right)dt$$

where *h*(*x*,*t*) is the transverse displacement of the body mentioned above. The dimensionless power coefficient (*C_P_*) can also be given as follows:

(8) $$C_{P}=\frac{2P_{in}}{\rho U^{3}L}$$

And the useful power (*P_u_*) is stated as below:

(9) $$P_{u}=\frac{1}{T}\int_{0}^{T}F_{x}\left(t\right)Udt$$

The propulsive efficiency parameter is expressed as follows:

(10) $$\eta =\frac{P_{u}}{P_{in}}=\frac{C_{T}}{C_{P}}$$

This parameter is examined to evaluate the relationship between the ability of the fish to produce thrust and the energy spent to push the water [33].

### 2.5. Prediction Model

**Figure 6:**
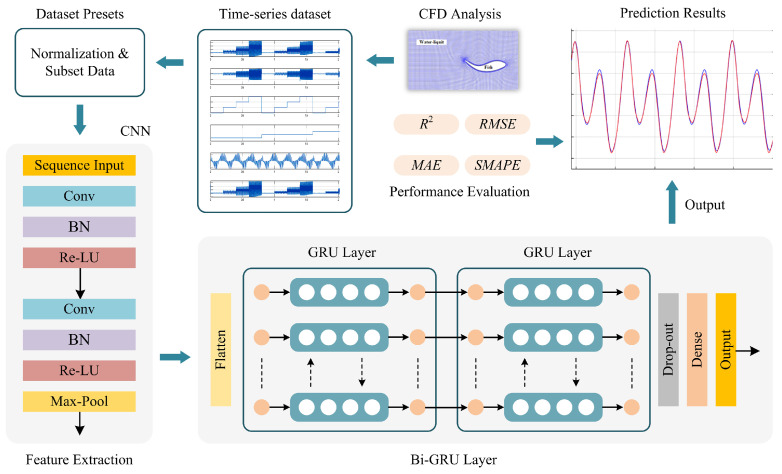
Overall scheme of the proposed prediction approach.

The main objective of the prediction model is to investigate the efficiency of an ensemble architecture combining deep learning-based models. Figure 6 shows the network structure of the proposed prediction approach. From the CFD analysis, the obtained hydrodynamics data are transferred to the deep learning model. In this model, the CNN-GRU structure is designed. The designed network consists of twelve layers with two convolution layers with batch-normalization (BN) and Re-LU, one max-pooling, one flatten, two GRU layers with a drop-out, and one dense layer, respectively. While the CNN structure extracts the distinctive features, the GRU structure effectively predicts the thrust force [34,35]. In the convolutional network, the connections are given as *Conv*(3, 1, 64)–*BN*–*Re-LU*(0.01)-*Conv*(3, 1, 32)–*BN*–*Re-LU*(0.01)-*Max*-*Pool*(2). In this design, the coarse grid search is applied to explore filter sizes with (16, 32, 64, 128), GRU unit numbers with (16, 32, 64), and dropout rates with (0.1, 0.25, 0.3, and 0.5), randomly. The optimal configuration is found to consist of 64 filters in the first convolution layer, 32 filters in the second layer, (64, 32) LSTM and GRU units, and a dropout rate of 0.25, yielding a strong balance between accuracy and generalization. After these operations, the extracted features are fed to the GRU structure. According to the above expressions, the *Conv*(·) layers compute the output of the neurons with filters to generate the feature maps. The convolution operation can be given by the following:

(11) $$y_{n,j}=f\left(\sum_{n=1}^{k-1}I_{n\;}*{\;W}_{n,j}+a_{j}\right)$$

where f(⋅) is the activation function and $a_{j}$ is the adding bias vector of the *j*th neuron. The I and W represent the input of the convolution and weight matrix.

**Figure 7:**
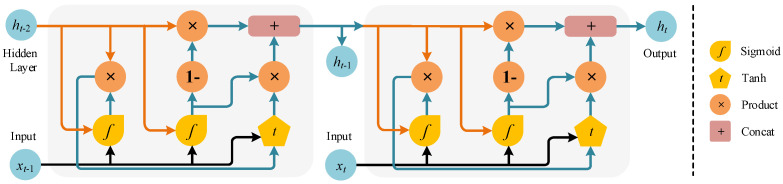
Bi-GRU structure.

The GRU is a recurrent neural network that is composed of update ($U_{t}$) and reset ($R_{t}$) gates. For an $x_{t}$ input, the general Bi-GRU structure used in this study is given in Figure 7. While the $U_{t}$ determines the activity with the previous cell, the $R_{t}$ is calculated as follows:

(12) $$U_{t}=\sigma \left(W_{i}^{U}\left[x_{t},h_{t-1}\right]+b_{i}^{U}\right)$$

(13) $$R_{t}=\sigma \left(W_{i}^{R}\left[x_{t},h_{t-1}\right]+b_{i}^{R}\right)$$

(14) $${\hat{h}}_{t}=tanh\left(Wx_{t}+R_{t}\;oWh_{t-1}\right)$$

(15) $$h_{t}=U_{t}\;o{\;h}_{t}-1+\left(1-U_{t}\right)o{\hat{\;h}}_{t}$$

here, $h_{t-1}$ is the cell state, W is the weight vector of the associated gate, σ is the sigmoid function, and o is the Hadamard product, respectively. In the experiments, CNN, LSTM, GRU, and variations of these models, such as CNN-LSTM and CNN-GRU, are analyzed.

### 2.6. Prediction of the Thrust Force

In these experiments, the dataset collected from the CFD analysis consists of 80.000 samples in total. The dataset contains the flapping frequency, flow velocity, thrust force, and lateral force, respectively. While the flapping frequency, flow velocity, and lateral force are selected as the input variables, the thrust force is the output. In addition, the two delayed thrust forces are also added to the input data to increase the model performance. For the prediction process, all data for the 0.3 m/s flow velocity experiments are separated as test data, and 80% of the remaining data are selected for training, with 20% for validation. In addition, the training and validation data are shuffled to overcome the negative effect of overfitting. Therefore, a robust decision-making performance can be obtained. Before training, all input and output variables are normalized using the min–max scaling method. This normalization ensures that all features are mapped into a stable numerical range, allowing the CNN and GRU models to operate effectively. Before performance evaluation, thrust force is also denormalized using the corresponding min–max parameters to obtain physically meaningful outputs. In the training phase, the mini-batch size is given as 128. The initial learning rate is selected as 0.01, and it is reduced with a drop factor of 0.5 at every 10 epochs. The maximum epoch is also set to 50. For all network models, the whole cost function is optimized with the adaptive moment estimation (Adam) algorithm during the backpropagation.

### 2.7. Evaluation Metrics

In order to evaluate the quantitative performance of the deep learning models, four performance metrics are statistically calculated: the coefficient of determination (*R*^2^), root mean square error (*RMSE*), mean absolute error (*MAE*), and symmetric mean absolute percentage error (*SMAPE*). *R*^2^ defines the proportion at which the change in the dependent variable is predicted from the independent variables. *RMSE* is the standard deviation between the actual and predicted data. While *MAE* gives the average magnitude of the error, *SMAPE* measures the accuracy based on the relative errors. These metrics can be expressed with the below equations:

(16) $$R^{2}=1-\frac{{\sum_{k=1}^{N}\left(X_{k}-Y_{k}\right)}^{2}}{{\sum_{k=1}^{N}\left(X_{k}-{\bar{X}}_{k}\right)}^{2}}$$

(17) $$RMSE=\sqrt{\frac{1}{N}\sum_{k=1}^{N}{\left(X_{k}-Y_{k}\right)}^{2}}$$

(18) $$MAE=\frac{1}{N}\sum_{k=1}^{N}\left|X_{k}-Y_{k}\right|$$

(19) $$SMAPE=\frac{1}{N}\sum_{k=1}^{N}\frac{\left|X_{k}-Y_{k}\right|}{\left(X_{k}-Y_{k}\right)/2}$$

where $X_{k}$ and $Y_{k}$ are the actual and predicted values, N is the sample size, and ${\bar{X}}_{k}$ is the mean of the actual value.

## 3. Results and Discussions

### 3.1. Hydrodynamic Analysis

**Figure 8:**
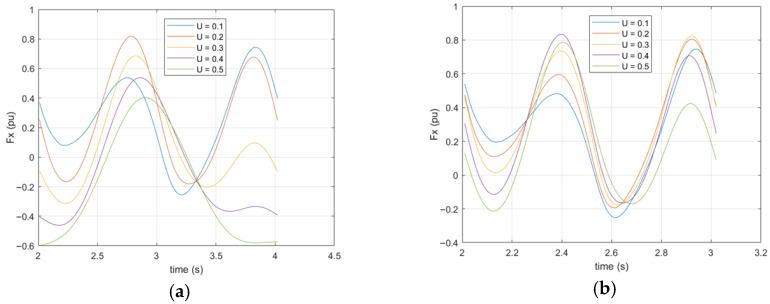

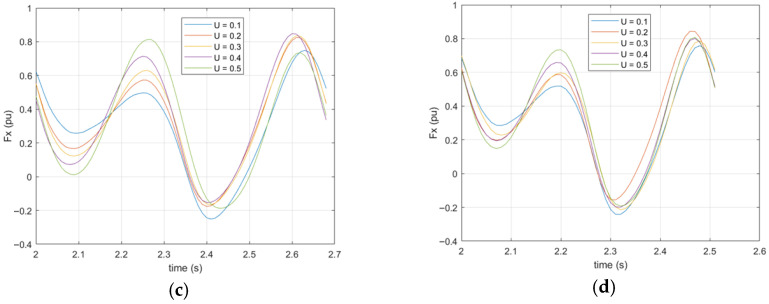
Hydrodynamic forces for a period of body motion: (**a**) *f* = 0.5 Hz, (**b**) *f* = 1 Hz, (**c**) *f* = 1.5 Hz, and (**d**) *f* = 2 Hz.

The Reynolds number (*Re*) is an important parameter to understand the behavior of fluids, to determine the flow regimes, and to see the behavior of the soft robot model. In this study, the interaction between *Re* and the drag force is investigated by changing the flow velocity in the range of [0.1, 0.5] (m/s), with a regular increase (0.1 m/s) for four different frequencies. The Reynolds number is calculated for flow speeds between 0.1 m/s and 0.5 m/s using the length *L* = 1 m and the kinematic viscosity of water *ν* = 1.0038 × 10^−6^ m^2^/s. The *Re* values corresponding to flow velocities are presented in Table 3. With the periodic oscillation movement of the fish body at a certain frequency, the resulting hydrodynamic forces show periodic fluctuations, as shown in Figure 8.

From Figure 8, it is seen that as the flow velocities and therefore the *Re* increase; the thrust forces increase because more fluid is moved, and this provides a higher momentum change. Since the increase in the flow rate of the fluid increases the lateral force acting on the fish body, there may be distortions in the thrust force that provide the forward movement of the fish at low fish speeds (see Figure 8a). As the forward speed of the fish increases due to the flapping frequency (*f*), the increase in the flow velocity of the fluid does not cause distortions in the thrust force (see Figure 8b–d).

Many studies in the literature have shown that the most appropriate way to discuss the effect between the oscillatory movement of fish in water and the flow rate is to examine the Strouhal number (*St*). The Strouhal number (*St*) specified in Equation (4) represents the ratio of the oscillation speed to the forward speed. However, some points should be taken into consideration when examining the effect of *St* on propulsive swimming performance, as stated in reference [36]. If different values of the flow velocity are taken into account to reach different values of *St*, this also means that the *Re* expressed by Equation (3) also changes. In this case, it becomes difficult to see the clear effect of the change in the *St* number on the propulsive performance. In addition, a high *Re* directly affects the vortex structures and distribution in the flow field around the moving part.

**Figure 9:**
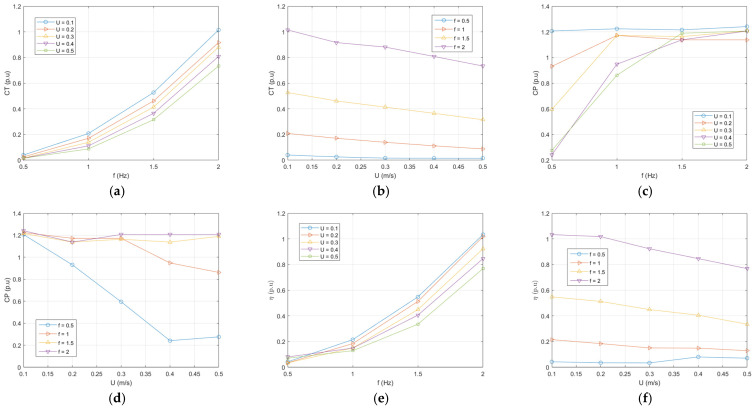
Variation in (**a**) thrust force coefficients for frequencies at various flow velocities, (**b**) thrust force coefficients for flow velocities at various frequencies, (**c**) power force coefficients for frequencies at various flow velocities, (**d**) power force coefficients for flow velocities at various frequencies (**e**) efficiency for frequencies at various flow velocities, and (**f**) efficiency for flow velocities at various frequencies (frequencies and flow velocities are determined as [0.5, 1, 1.5, 2] (Hz) and [0.1, 0.2, 0.3, 0.4, 0.5] (m/s), respectively).

Considering the flapping frequency values of the analyzed fish body and the flow velocity values of the fluid within the scope of this study, the obtained *St* numbers are presented in Table 4. Thrust force coefficients (*C_T_*), power force coefficients (*C_P_*), and propulsive efficiency (*η*), which are expressed in Equations (6), (8), and (10) for four different frequency values, are evaluated at different flow velocities in Figure 9.

Fish increase their flapping frequency and flapping amplitude to increase forward speed. Increasing the frequency causes an increase in the thrust force and increases the *C_T_* ratio. These results can be observed simultaneously in Figure 9a,b and are also confirmed by Equations (5) and (6). In Figure 9a, *C_T_* curves with similar slopes are observed with increasing flow rate values, with an equal increase amount. However, while increasing the frequency increases *St*, increasing the flow rate decreases *St* and at the same time increases Re, which changes the structure of the flow. In other words, the fact that *St* and *Re* affect the propulsive performance together, which we mentioned above, is also understood from the slope difference when Figure 9a,b are considered. In Figure 9b, the same flapping frequency and the corresponding increase in flow velocity at the same forward propulsive speed value do not change the *C_T_* ratio very much. The rate of change in *C_T_* increases slightly as the flapping frequency increases. It is also observed from Figure 9b and Equations (5) and (6) that *C_T_* decreases as the flow rate increases. Because the frequency is constant, there is no change in the thrust force, which causes the *C_T_* ratio to tend to decrease as the flow velocity increases.

As the flapping frequency increases, which increases the fish’s propulsive force, this means more input power and increases the power coefficient. Figure 9c confirms this finding. At a constant thrust force, an increase in the flow velocity does not change *C_P_* much at high thrust forces. This aligned with the findings reported by [36]. Since low thrust forces mean a low input power, increasing the flow velocity simultaneously implies a reduction in the power coefficient (Equation (8)). This situation is also shown in Figure 9d.

**Figure 10:**
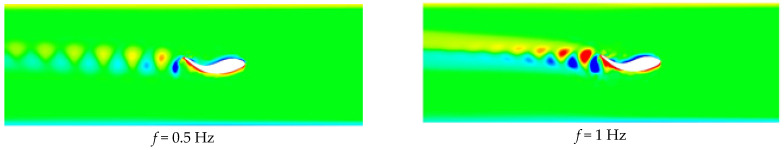

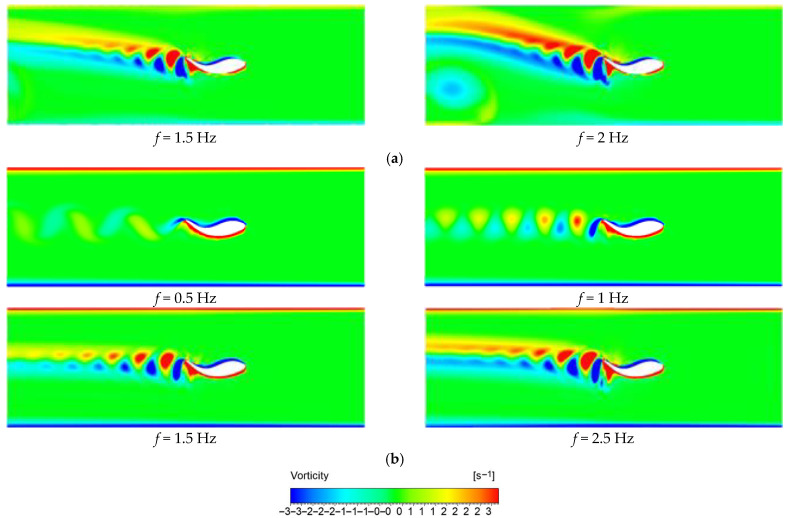
Vorticity patterns of the oscillation motion by the fish-like locomotion at (**a**) *U* = 0.2 m/s and (**b**) *U* = 0.5 m/s for different flapping frequencies (*t* = *T*).

Propulsive efficiency is obtained from the ratio of the obtained thrust force coefficient and power coefficient, which is proportional to the input power, as expressed in Equation (10). The propulsive efficiency increases with increasing thrust force and remains approximately constant with increasing flow velocity. Figure 9e,f confirm these findings. Considering the vorticity model generated in the water by the periodic oscillation movement of the fish, the vorticity contours obtained as a result of the oscillation motion of the fish at different flapping frequencies are displayed collectively in Figure 10a,b. An analysis at various flow rates is included in Appendix A. When the fish body moves in waves, the shear layer along the body separates from the end of the tail, leading to the shedding of vortices. From Figure 10, it is seen that, as the frequency of the oscillation motion and hence St increase in the case where *Re* is a constant, the vorticity intensity increases. In the low flapping frequency of the fish body (*f* = 0.5 Hz), periodic vortices are seen, forming a repeating pattern. This pattern can be expressed as the known von Karman vortex street.

**Figure 11:**
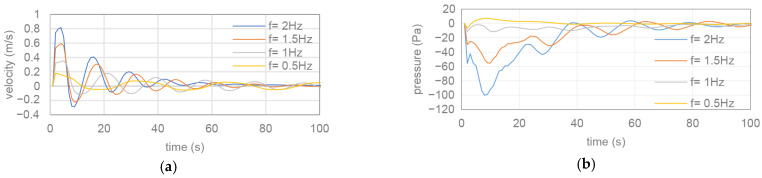
(**a**) The background flow velocities and (**b**) pressures for determined flapping frequencies.

Additionally, at high flapping frequencies, the number of vortices shed from the tail increases, and the resulting vortex traces become more chaotic. As the *St* number increases with the increase in the frequency of the oscillation motion, it is observed that vortex columns with two layers are formed. This shows that the *St* number greatly affects the formation and arrangement of the vortices. As seen in Figure 10, when the vortex contours become thinner at low flow velocities, it is observed that the vortices produced by the flapping motion are deflected towards the upper part of the flow region. As the flow velocity increases, the vortex distribution evolves to be located in a straight line towards the fish axis. The vortex structures obtained from the simulation results are quite consistent with the results reported by Guo et al. and Ghommem et al. [37,38]. When these vortex structures and thrust efficiency (η) trends are evaluated together, it is seen that the increase in the *St* number, in particular, is a determining factor in propulsive performance. The single-row and regular von Karman vortex pattern that occurs at low frequencies allows the flow to carry more stable forward momentum and limits energy loss. In contrast, the double-row vortex structure that forms at high *St* values creates a higher vortex density and more complex flow separation at the tail end, causing some of the energy to dissipate laterally. This leads to increased fluctuations in thrust force over time and a limitation of the efficiency beyond a certain level. Therefore, the vortex pattern observed in Figure 10 provides a physical explanation consistent with the efficiency trends in Figure 9. The effects of tail frequencies on the background flow velocity and pressure are given in Figure 11.

As shown in Figure 11, both the forward velocities and pressures are in agreement with the background vorticity patterns. These results collectively indicate a strong coupling between the flapping frequency, flow velocity, Reynolds number, and Strouhal number. Increasing the flapping frequency enhances thrust generation while simultaneously altering vortex dynamics, leading to more chaotic and dense vortex structures at higher *St* numbers. In contrast, low-frequency oscillations produce stable and periodic von Kármán vortex streets. Additionally, the increase in flow velocity realigns vortex structures along the swimming direction, demonstrating how the Reynolds number modifies vortex organization. These hydrodynamic behaviors provide essential insights into the mechanisms governing propulsive efficiency and thrust generation in biomimetic underwater propulsion systems.

Although the validation steps presented in this study are performed using theoretical thrust and power coefficients, a direct comparison with experimental thrust measurements in the literature (e.g., Li et al. [18]) is not possible due to some limitations of the current model. Directly matching the 2D CFD model with 3D experimental data is methodologically inappropriate, as parameters such as the 3D geometry of the fish body, tail stiffness, propulsion mechanism, and flow field dimensions do not correspond exactly to the physical prototypes in the literature. However, the consistent behavior of the hydrodynamic coefficients obtained under different flapping frequency and flow velocity conditions supports the reliability of the model. In future studies, we plan to produce a soft robotic tail prototype compatible with the geometry of the current fish model, conduct direct thrust measurements in a laboratory environment, and compare these experimental data with CFD results.

### 3.2. Prediction Results

**Figure 12:**
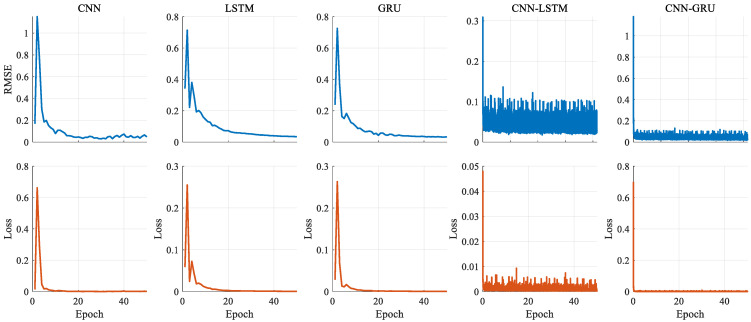
Training progresses of the deep learning models.

**Figure 13:**
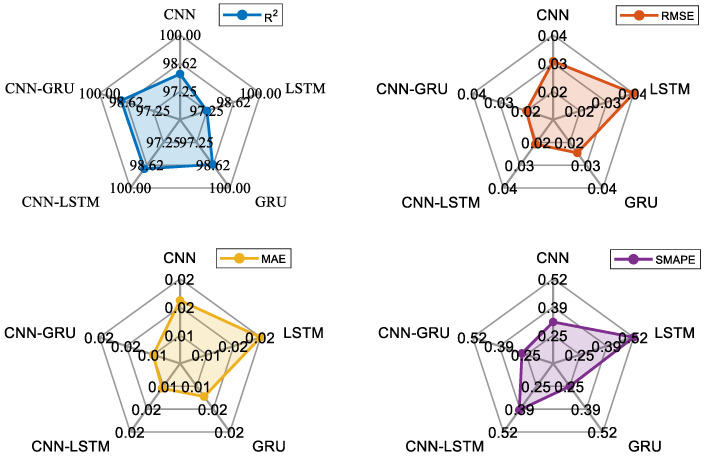
Performance metric presentation of the deep learning models.

To illustrate the learning dynamics, the training processes of all models are given in Figure 12. These curves indicate a smooth convergence, with a loss after approximately 20 epochs, and the best smooth behavior is observed with CNN-GRU. The consistency between the *RMSE* and loss curves confirms the robustness of the training procedures. The performance metric results of the deep learning models are given in Table 5. According to Table 5, the *R*^2^ value of the CNN-GRU is obtained as 0.9889, while the other deep learning models are 0.9810 for CNN, 0.9725 for LSTM, 0.9858 for GRU, and 0.9884 for CNN-LSTM, respectively. When all deep learning models are analyzed regarding their *RMSE* and *MAE* values, the CNN-GRU gives the best values, which are 0.0228 and 0.0103. For these values, CNN, LSTM, GRU, and CNN-LSTM are calculated as being 0.0298 and 0.0174, 0.0359 and 0.0218, 0.0258 and 0.0129, and 0.0233 and 0.0108, respectively. However, the CNN-GRU model gives the second-best *SMAPE* value, with 0.2727. While the lowest *SMAPE* is achieved with the GRU model, at 0.2505, the highest is obtained at 0.5241 with the LSTM model. According to all metrics, a general comparison is also given in Figure 13 to show the performance order of the models. From these values, it can be clearly seen that using a hybrid structure with combined CNN-LSTM and CNN-GRU models increases the prediction performance through the ability of the CNN structure to extract distinctive features. However, the LSTM has a separate update gate and forget gate, making the CNN-LSTM more complex than the CNN-GRU model. The all-metric results of the GRU-based model improve compared to those of the LSTM-based model. As a result, the designed CNN-GRU model can make significant contributions to prediction success, with the highest performance and simpler model architecture.

**Figure 14:**
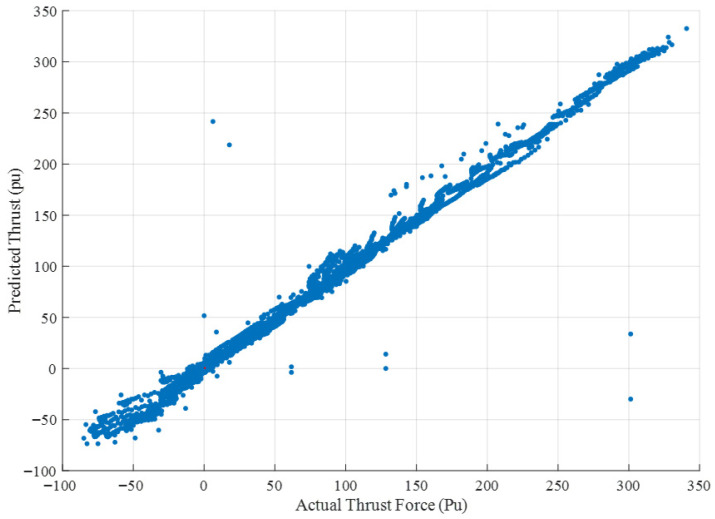
Scatter plots of predicted versus actual thrust force (pu) for the CNN-GRU model across all frequency cases.

In addition, all force-related quantities, including the *RMSE* and *MAE*, were expressed in per-unit (pu) format for a consistent comparison across different operating conditions. The thrust force was normalized by the maximum value observed in the CFD simulations. Therefore, the *RMSE* value of the best model of 0.0228 pu corresponds to 2.28% of the maximum thrust value. This representation provides meaningful scaling and enables a direct comparison of prediction accuracy across models. A scatter graph of predicted versus actual thrust force values (pu) for the CNN-GRU model is given in Figure 14. Most points tightly follow the 45° reference line, demonstrating an excellent prediction accuracy. A small cluster of points with slightly higher deviation appears, corresponding to highly unsteady high-frequency flapping conditions. The small group of points represents flow states where the tail motion generates stronger vortex structures. These unsteady transitions introduce mild prediction variability, which is expected due to the increased nonlinearities in the hydrodynamic field at high thrust amplitudes. These deviations remain minor and are physically consistent with the increased flow complexity at high thrust levels.

To more comprehensively address the success of the deep learning models in the prediction of *Fx* from the hydrodynamics analysis, the experiments are also divided into four frequency cases as 0.5 Hz, 1 Hz, 1.5 Hz, and 2 Hz. Therefore, the performance and reliability of the CNN-GRU model are analyzed in all frequency variations. In these analyses, the frequency intervals are determined from the testing data, and predictions are given for all models. Specific sample intervals are also visualized to better show how the deep learning models track actual force data.

**Figure 15:**
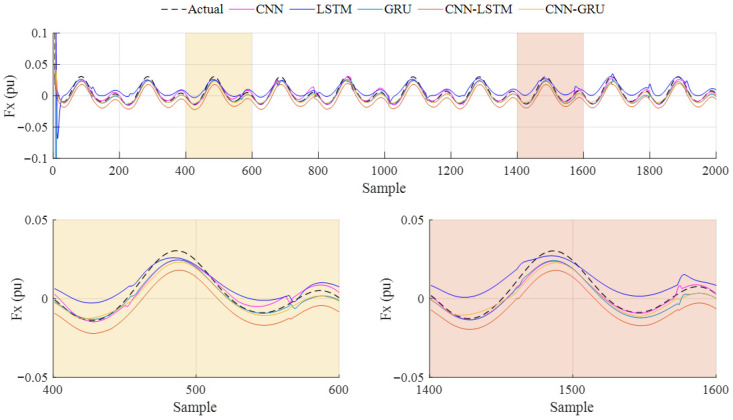
Case-1: Prediction results of the deep learning models for 0.5 Hz flapping frequency.

The prediction results of Case-1 are presented in Figure 15. In addition, the performance metric results obtained from all methods are given in Table 6. According to these results, the highest *R*^2^ and *RMSE* values are obtained with the CNN. While the *R*^2^ and *RMSE* of the CNN-GRU are 0.9054 and 0.0040, the lowest *R*^2^ is shown with the LSTM, at 0.6490, and the highest *RMSE* is shown with the CNN-LSTM, at 0.0230. When the *MAE* and *SMAPE* values are compared, the lowest values are obtained with the CNN. The *MAE* and *SMAPE* of the CNN-GRU model are calculated as being 0.0034 and 0.5905. From these results, the CNN model provides better prediction results than the other models for Case-1.

**Figure 16:**
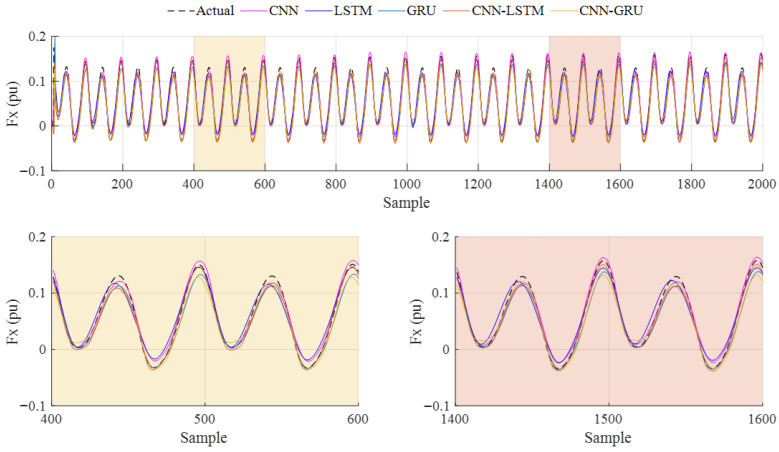
Case-2: Prediction results of the deep learning models for 1 Hz flapping frequency.

The prediction results obtained from Case-2 are given in Figure 16. The performance metric results are summarized in Table 6, and the following evaluations can be seen. When the *R*^2^ and *RMSE* values are analyzed, which are 0.9763 and 0.0089, the CNN model gives the better performance. While the lowest *MAE* is obtained with the CNN-LSTM, at 0.0061, the lowest *SMAPE* is achieved with the GRU, at 0.2126. These results show that the CNN model achieves better *R*^2^ values at low flapping frequencies, but, when all metric results are evaluated, the models give different prediction performances.

**Figure 17:**
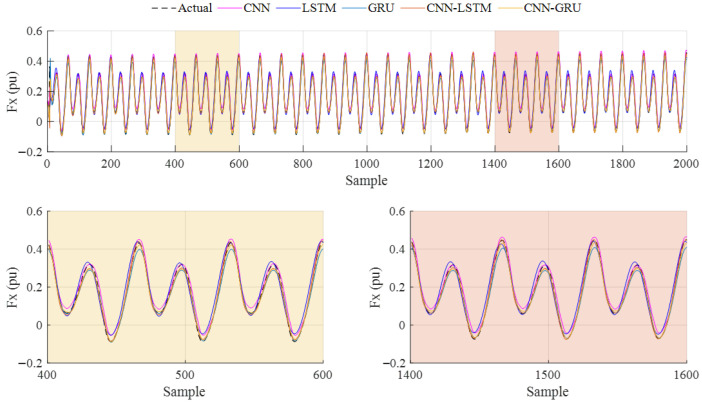
Case-3: Prediction results of the deep learning models for 1.5 Hz flapping frequency.

In order to further analyze the prediction accuracy and effectiveness of the deep learning models at higher frequency values, the comparison of Case-3 is carried out. The obtained results are given in Figure 17, and the metric results for this case are listed in Table 6. When the *R*^2^ and *RMSE* values are analyzed, which are 0.9879 and 0.0163, the CNN-GRU model has the better values among the other models. The LSTM model presents the worst values, at 0.9559 and 0.0312. According to the *MAE* and *SMAPE* metric values, the CNN-GRU model obtains the lowest results, which are 0.0102 and 0.1147. The second-best performance of all metric results is achieved with the CNN-LSTM, with the results calculated as being 0.9866, 0.0172, 0.0106, and 0.1167, respectively.

**Figure 18:**
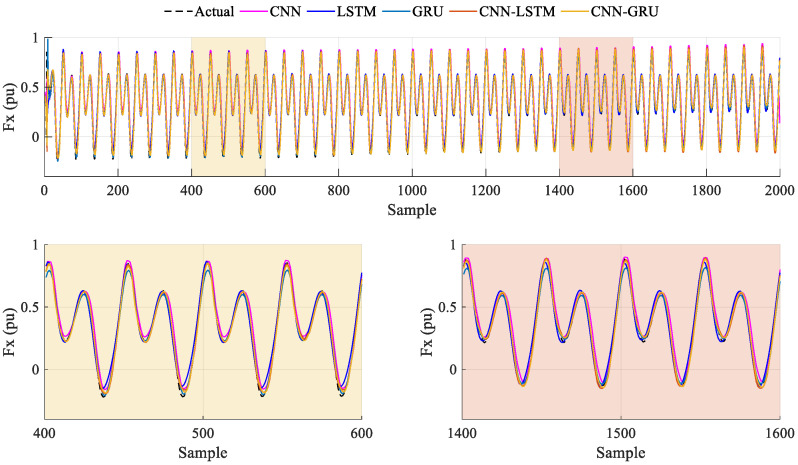
Case-4: Prediction results of the deep learning models for 2 Hz flapping frequency.

**Figure 19:**
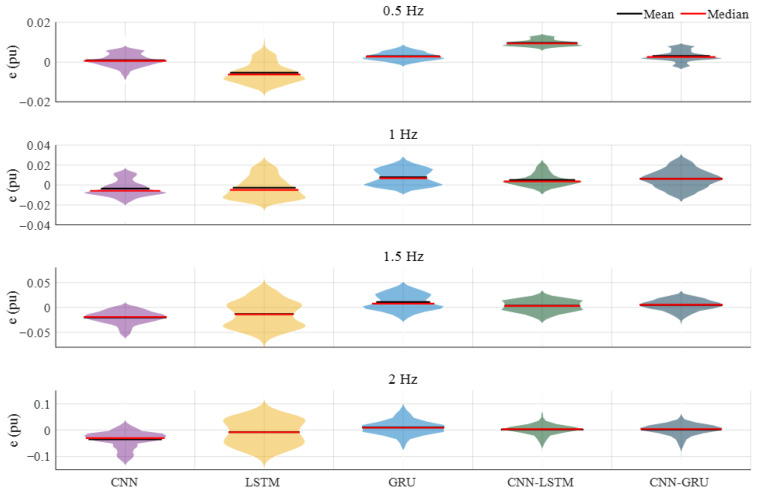
Prediction errors of the deep learning models.

Figure 18 shows the prediction performance of Case-4. According to these results, the CNN-LSTM and CNN-GRU models give the highest *R*^2^ value, at 0.9860. While the lowest *RMSE* and *MAE* values are achieved at 0.0353 and 0.0162 with the CNN-LSTM model, the second-lower values are obtained with the CNN-GRU, at 0.0354 and 0.0178. For the SMAPE values, the CNN-GRU gives the lowest value, at 0.0978, while the CNN-LSTM achieves a 0.1097 value. In these evaluations, the LSTM model exhibits the worst performance for all metrics. As can be seen from these comprehensive results, the CNN-LSTM and CNN-GRU models exhibit a higher prediction performance with increasing flapping frequency, and the CNN-GRU demonstrates a superior prediction performance in the overall evaluation. In Figure 19, the violin plots are given to show and compare the prediction error distribution for all frequency variations. Similarly to the above comparisons, the mean and median error values of the CNN-GRU model are close to the zero point, and the distribution area decreases when the frequency is increased. It is clearly seen that accurate and sensitive prediction results can be obtained with the CNN-GRU, and it can guarantee satisfactory and high performance for *Fx* prediction from hydrodynamics analysis.

As the CNN-GRU model shows a superior performance in the experiments, the improvement percentages of the other models compared to the CNN-GRU are presented in Table 7. Accordingly, the GRU-based model using convolutional layers as the feature extraction shows a remarkable level of improvement compared to other models, which proves that the prediction ability of the CNN-GRU is superior to other methods. Compared to the *R*^2^ values of CNN, LSTM, GRU, and CNN-LSTM, the CNN-GRU improves the results between 0.05 and 1.69%. For the *RMSE* values, the highest improvement is seen in the LSTM, with 36.49%, and the lowest improvement is seen in the CNN-LSTM, with 2.15%. Among all metrics, the highest improvement is seen in the LSTM model with a 52.75% *MAE* value. This value is the lowest in the CNN-LSTM model, with 4.63%. For the *SMAPE* performance, the CNN-GRU improves the GRU model by 8.86%. The highest improvement is in the LSTM model, with 47.97%. These results prove that the CNN-GRU-based approach provides a more accurate performance in the prediction than other models.

The prediction results further show that hybrid deep learning architectures offer significant advantages in modeling hydrodynamic force patterns. The CNN-GRU model outperforms other architectures because the CNN layers effectively extract spatial flow features, while the GRU units capture temporal dependencies in oscillatory thrust generation. The superior performance at higher flapping frequencies indicates that the GRU structure more effectively models rapidly varying force dynamics compared to the LSTM, which suffers from parameter complexity and gate saturation. These findings highlight how data-driven modeling can complement CFD analyses by providing a fast and accurate estimation of hydrodynamic forces under varying kinematic conditions.

Although the used dataset consists exclusively of 80.000 CFD-generated samples, the proposed deep learning framework is designed to be extendable to real experimental conditions. The CFD environment provides practical hydrodynamic data, enabling the model to learn physically consistent thrust force patterns across varying flapping frequencies and flow velocities. However, differences in the physical environments may limit generalization. To overcome this limitation, the developed CNN-GRU architecture can be adapted when experimental measurements become available. Specifically, the convolutional layers responsible for extracting low-level flow features can be retained, while the GRU layers can be fine-tuned using a set of real sensor data. This approach would allow the model to generalize for unseen configurations and improve the prediction reliability in a real robotic fish system.

## 4. Conclusions

In this study, various factors affecting the underwater performance of the fish model are investigated. For instance, the relationship between the Reynolds number and flapping frequency is significant. Flow velocities are studied between 0.1 m/s and 0.5 m/s with increasing speeds in a certain order, and flapping frequencies are changed for each speed to observe the effects on the thrust force, power coefficient, and propeller efficiency. The obtained data show that, as the flow velocity increases, the thrust force also increases because more fluid is moved. This also leads to a change in momentum. However, at low velocities, deteriorations in thrust force are observed due to the increase in horizontal forces; however, these deteriorations could be prevented by increasing the flapping frequency.

The Strouhal number (*St*) plays a significant role as a key parameter explaining the performance of fish movements in water. In the study, Strouhal numbers obtained with different flow velocities revealed that the Reynolds number increases as the flow velocity increases, and this increase changes the flow structure. This interaction has been a key factor in comprehending the relationship between the thrust coefficient (*C_T_*) and the power coefficient (*C_P_*). The study confirms that increasing the frequency increases the thrust force and, as a result, the power coefficient also increases. The effect of the flow rate is seen more clearly, especially at low thrust forces, in which case it is understood that the increase in the flow rate decreases the power coefficient. The propulsive efficiency (*η*) is defined as the ratio of the thrust force to the input power, and, in this study, it is observed that the efficiency increased in direct proportion to the thrust force. The increase in the flow velocity did not result in a major change in efficiency. This shows that the thrust force reaching a certain level does not affect the efficiency more. In addition, the vortex structures created by the periodic oscillation movement of the fish’s body are also examined. While the vortices formed owing to high-frequency flapping become more chaotic and dense, a more regular vortex pattern is observed at low frequencies. As the flow rate increased, these vortices were arranged more straightly, parallel to the movement direction of the fish. These results provide important data to understand the structure of the flow around the fish and the development of vortices.

The dataset obtained from the CFD analysis consists of 80,000 samples, including flapping frequency, flow velocity, thrust force, and lateral force. Flapping frequency, flow velocity, and lateral force are used as input variables, while the thrust force is the output. Additionally, two delayed thrust forces are added to improve the model performance. The data from experiments conducted with a 0.3 m/s flow velocity are used as test data, with 80% of the remaining data used for training and 20% for validation. To prevent overfitting, the training and validation data are shuffled. The performance of the deep learning models is evaluated using four metrics: *R*^2^, *RMSE*, *MAE*, and *SMAPE*. The CNN-GRU model achieved the highest *R*^2^ value of 0.9889, outperforming CNN (0.9810), LSTM (0.9725), GRU (0.9858), and CNN-LSTM (0.9884). CNN-GRU also produced the best *RMSE* (0.0228) and *MAE* (0.0103) values. While the lowest *SMAPE* value (0.2505) was obtained by the GRU model, the LSTM model had the highest *SMAPE* value (0.5241). Overall, combining CNN and GRU models into a hybrid structure improved the prediction performance.

The analysis of the experiments at different frequencies (0.5 Hz, 1 Hz, 1.5 Hz, and 2 Hz) showed that the CNN model performed best at 0.5 Hz, while CNN-GRU performed better at 1 Hz and has a superior prediction accuracy at higher frequencies (1.5 Hz and 2 Hz). The CNN-GRU model demonstrated a superior performance compared to the other models, with improvements in *R*^2^ values ranging from 0.05% to 1.69%. The largest improvement in *RMSE* and *MAE* is observed in the LSTM model (36.49%). These results confirm that the CNN-GRU model provides more accurate predictions than the other models.

In conclusion, this study provides a deeper understanding of the complex interactions between the flapping frequency, flow velocity, Reynolds number, and Strouhal number, thereby optimizing the performance of underwater biomimicry-based robots. The correct analysis of these interactions can serve as a crucial guide in the design of fish-like underwater propulsion systems. This study employs a two-dimensional CFD model, which is widely used for the preliminary analysis of undulatory locomotion but inherently neglects spanwise vortex interactions and tip-vortex formation. These 3D flow structures can significantly influence thrust generation, lateral forces, and vortex stability, especially at higher Reynolds numbers or for fish with a pronounced body depth. Future work will therefore focus on extending the present framework to a fully 3D CFD domain, which is expected to provide more realistic vortex structures, an improved prediction of propulsive efficiency, and more accurate coupling with deep learning models. Although 2D analysis offers a reduced computational cost and sufficiently captures the primary oscillatory thrust mechanisms, a 3D analysis will allow a more comprehensive representation of the physical behavior of biomimetic fish swimming. In the future study, the force effects of the soft robotic tail at different flapping frequencies will also be investigated in a real pool environment. The study will be presented in comparison with the results obtained from both 2D and 3D CFD analysis. Furthermore, a future objective is to develop an underwater prediction methodology by classifying the vortex signatures produced by fish exhibiting different body structures and tail shapes.

## Figures and Tables

**Table 1:** Comparison of CFD–ML/CFD-assisted biomimetic fish studies and this work.

Feature	Representative CFD–ML Studies (e.g., ref. [4])	CFD-Assisted Biomimetic Fish Studies [7,8,9,10,11,12,13,14,15,16,17,18,19,20,21,22,23,24,25,26,27,28,29]	This Work
Geometry	2D NACA0012 airfoil	Mostly rigid or simplified; some soft robotic prototypes	Soft-bodied 2D fish with deformable tail
Motion Model	UDF-driven full oscillatory self-propelled motion	CPG-controlled fins; undulating fins; schooling dynamics	UDF-driven full oscillatory self-propelled motion
CFD Method	2D RANS, overset dynamic mesh	2D/3D RANS; FSI-inspired models; vortex/wake analysis	2D RANS with fully dynamic mesh and vortex resolution
Use of ML/Optimization	MLP	Mostly CFD only; limited ML or optimization	Hybrid CNN–GRU (spatial + temporal feature extraction)
Outputs	Velocity, displacement, acceleration, St	Thrust, wake patterns, energy usage, fin hydrodynamics	Fx prediction at four flapping frequencies
Dataset Size	Small dataset	Case-based datasets	Large dataset (~80,000 samples)
Validation	Numerical only	CFD or experiment depending on study	Multi-frequency validation + *R*^2^, *RMSE*, *MAE*, *SMAPE*
Novelty	CFD–ML coupling	Hydrodynamic-focused; ML integration generally limited	CFD + hybrid deep model for vortex- induced force prediction

**Table 2:** Comparison of *C_D_* values corresponding to the mesh variable.

	Mesh Nodes	Mesh Elements	Drag Coefficient (*C_D_*)	Lift Coefficient (*C_L_*)	Orthogonal Quality Mesh Metrics (Min > 0.1)
M1	5447	5248	407.81445	156.66	0.68026
M2	8169	7929	445.65701	235.34	0.68943
M3	10,373	10,101	446.16295	275.78	0.64466
M4	14,039	13,730	462.82799	320.87	0.73006
M5	19,801	19,429	461.94348	318.73	0.69978
M6	30,543	30,083	463.97556	329.01	0.62281
M7	120,150	119,241	465.69931	330.14	0.59476

**Table 3:** The Reynolds numbers corresponding to the flow velocity variable.

*U* (m/s)	*Re*
0.1	99,621
0.2	199,242
0.3	298,863
0.4	398,484
0.5	498,105

**Table 4:** The examined Strouhal numbers.

*f* (Hz)	*U* (m/s)				
	0.1	0.2	0.2	0.4	0.5
	*St*				
0.5	0.5	0.25	0.15	0.125	0.1
1	1	0.5	0.3	0.25	0.2
1.5	1.5	0.75	0.45	0.375	0.3
2	2	1	0.6	0.5	0.4

**Table 5:** Performance metric results of the deep learning models.

Metric	CNN	LSTM	GRU	CNN-LSTM	CNN-GRU
*R* ^2^	0.9810	0.9725	0.9858	0.9884	0.9889
*RMSE*	0.0298	0.0359	0.0258	0.0233	0.0228
*MAE*	0.0174	0.0218	0.0129	0.0108	0.0103
*SMAPE*	0.3150	0.5241	0.2505	0.3931	0.2727

**Table 6:** Performance metric results of the deep learning models for frequency variations.

Metric	Frequency (Hz)	CNN	LSTM	GRU	CNN-LSTM	CNN-GRU
*R* ^2^	0.5	0.9369	0.6490	0.9258	0.6900	0.9054
	1	0.9763	0.9537	0.9510	0.9705	0.9536
	1.5	0.9724	0.9559	0.9769	0.9866	0.9879
	2	0.9694	0.9656	0.9819	0.9860	0.9860
*RMSE*	0.5	0.0033	0.0078	0.0036	0.0230	0.0040
	1	0.0089	0.0124	0.0128	0.0099	0.0124
	1.5	0.0246	0.0312	0.0225	0.0172	0.0163
	2	0.0522	0.0553	0.0401	0.0353	0.0354
*MAE*	0.5	0.0026	0.0066	0.0030	0.0102	0.0034
	1	0.0075	0.0099	0.0088	0.0061	0.0092
	1.5	0.0205	0.0250	0.0155	0.0106	0.0102
	2	0.0386	0.0437	0.0233	0.0162	0.0178
*SMAPE*	0.5	0.4375	1.1571	0.5505	1.1266	0.5905
	1	0.2904	0.3846	0.2126	0.2191	0.2881
	1.5	0.2875	0.2892	0.1255	0.1167	0.1147
	2	0.2429	0.2638	0.1088	0.1097	0.0978

**Table 7:** Improvement percentage results of the deep learning models.

Model	*R*^2^ (%)	*RMSE* (%)	*MAE* (%)	*SMAPE* (%)
CNN	0.81	23.49	40.80	13.43
LSTM	1.69	36.49	52.75	47.97
GRU	0.31	11.63	20.16	8.86
CNN-LSTM	0.05	2.15	4.63	30.63

**Figure A1:**
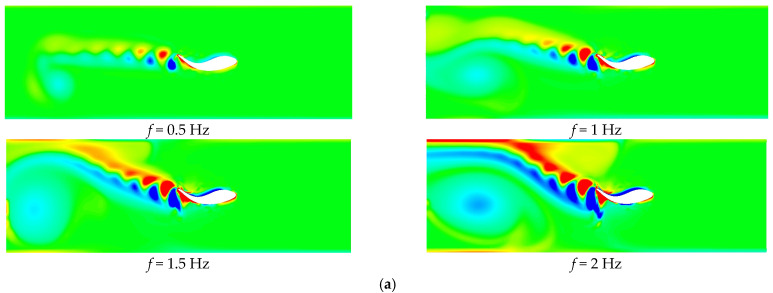

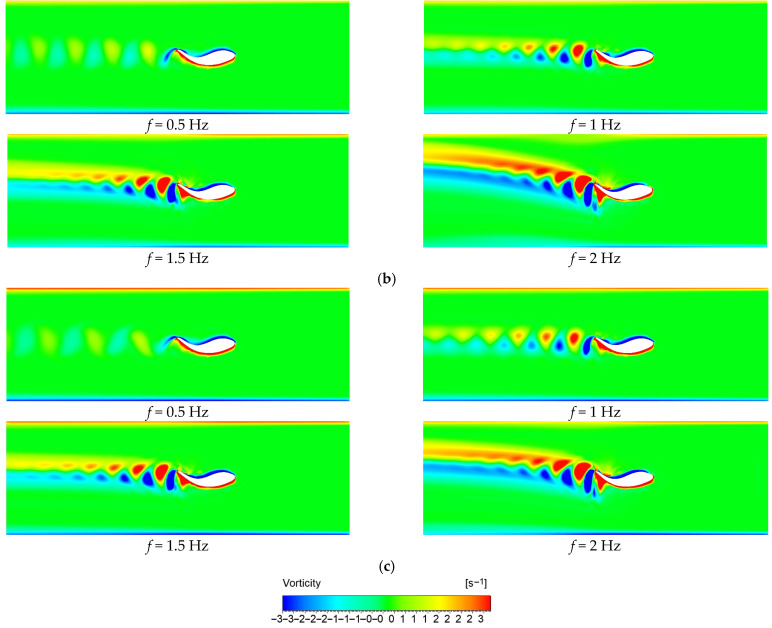
Vorticity patterns of the oscillation motion by the fish-like locomotion at (**a**) *U* = 0.1 m/s, (**b**) *U* = 0.3 m/s, and (**c**) *U* = 0.4 m/s for different flapping frequencies (*t* = *T*).

## Data Availability

The original contributions presented in this study are included in the article. Further inquiries can be directed to the corresponding author.

## Abbreviations

The following abbreviations are used in this manuscript:

AUV	Autonomous Underwater Vehicles
CFD	Computational Fluid Dynamics
LSTM	Long Short-Term Memory
CNN	Convolutional Neural Network
GRU	Gated Recurrent Network
TBF	Tail Beat Frequency
UDF	User-Defined Function
BN	Batch-Normalization
